# Venous gas caused by emphysematous pyelonephritis: a case report and review of literature

**DOI:** 10.1186/s12894-022-01104-6

**Published:** 2022-09-19

**Authors:** Xue Dong, Shuzong You, Huangqi Zhang, Dongnv Wang, Wenting Pan, Binhao Zhang, Shanqiang Huang, Xin Li, Jianxin Pang, Wenbin Ji

**Affiliations:** Department of Radiology, Taizhou Hospital, Zhejiang University, Taizhou, 318000 Zhejiang China

**Keywords:** Emphysematous pyelonephritis, Venous gas, *Escherichia coli*, Treatment, Pathogenesis

## Abstract

**Background:**

Emphysematous pyelonephritis (EPN) is a potentially life-threatening disease caused by a gas-producing necrotizing bacterial infection that involves the renal parenchyma, collecting system, and/or perinephric tissue. EPN is often complicated by a previous diagnosis of diabetes mellitus, and venous air bubbles are an uncommon complication of it. We describe a 52-year-old woman who was admitted in coma, with a history of vomiting, and was found to have EPN with air bubbles in the uterine veins. We discuss the presentation, diagnosis, and pathogenesis of this uncommon but clinically significant event, and briefly review other case reports of venous gas or thrombosis caused by EPN.

**Case presentation:**

We report the case of a 52-year-old woman with past history of type 2 diabetes mellitus, presenting with loss of consciousness after vomiting for half a day. Abdominal computed tomography scan revealed unilateral EPN with air bubbles in the uterine veins. The blood, pus, and urine cultures were positive for extended-spectrum beta-lactamase-producing *Escherichia coli*. The patient’s condition improved well after conservative management comprising supportive measures, broad-spectrum antibiotics, percutaneous drainage therapy, and an open operation.

**Conclusions:**

Venous air bubbles are rare but fatal complication of EPN. Early diagnosis and treatment are critical to ensure good results.

## Background

Emphysematous pyelonephritis (EPN) is a potentially life-threatening and acute, severe necrotizing bacterial infection. Its typical characteristics is that air is presented in the renal parenchyma, and para-renal space collecting system [1]. Venous gas is a rare complication of EPN. Here, we present a case of EPN with air bubbles in the uterine veins that was infected by extended-spectrum beta-lactamase-producing *E. coli.* In addition, we summarized other cases of EPN with venous gas or thrombosis. To our knowledge, this is the first report of EPN in which air bubbles were observed in the uterine veins.

## Case presentation

A 52-year-old Chinese woman was brought to the emergency room due to the history of worsening thirst, polydipsia, nausea and fatigue for 10 days and loss of consciousness after vomiting for half a day. When she was 40 years old, she was diagnosed with type 2 diabetes mellitus (T2DM), but she hesitated to receive any medical treatment.

On admission, she developed septic shock. Her temperature was 36.3 °C and blood pressure was 105/62 mmHg which was maintained with norepinephrine. She has tachypnea with respiratory rate of 25 breaths/min and rapid heart rate of 135 beats/min. The laboratory results showed a leukocytosis of 14.2 × 10^9^ per L with 95.8% neutrophils. Her procalcitonin concentration was severely increased at 170 ng/mL, and her blood lactate level was 4.8 mmol/L. She had abnormal renal function markers, including serum creatinine and urea nitrogen levels of 3.0 mg/dL and 17.7 mmol/L, respectively. She had hyperglycemia with a blood glucose concentration of 27.8 mmol/L. The patient was transferred to the intensive care unit. And she was treated with intravenous fluid, received norepinephrine, bowel rest, intravenous injection of small doses of insulin, and broad-spectrum antibiotics. However, septic shock was not improved and primary lesions leading to septic shock was unknown. Therefore, perform an urgent computed tomography (CT) scan to confirm the diagnosis.

**Fig. 1:**
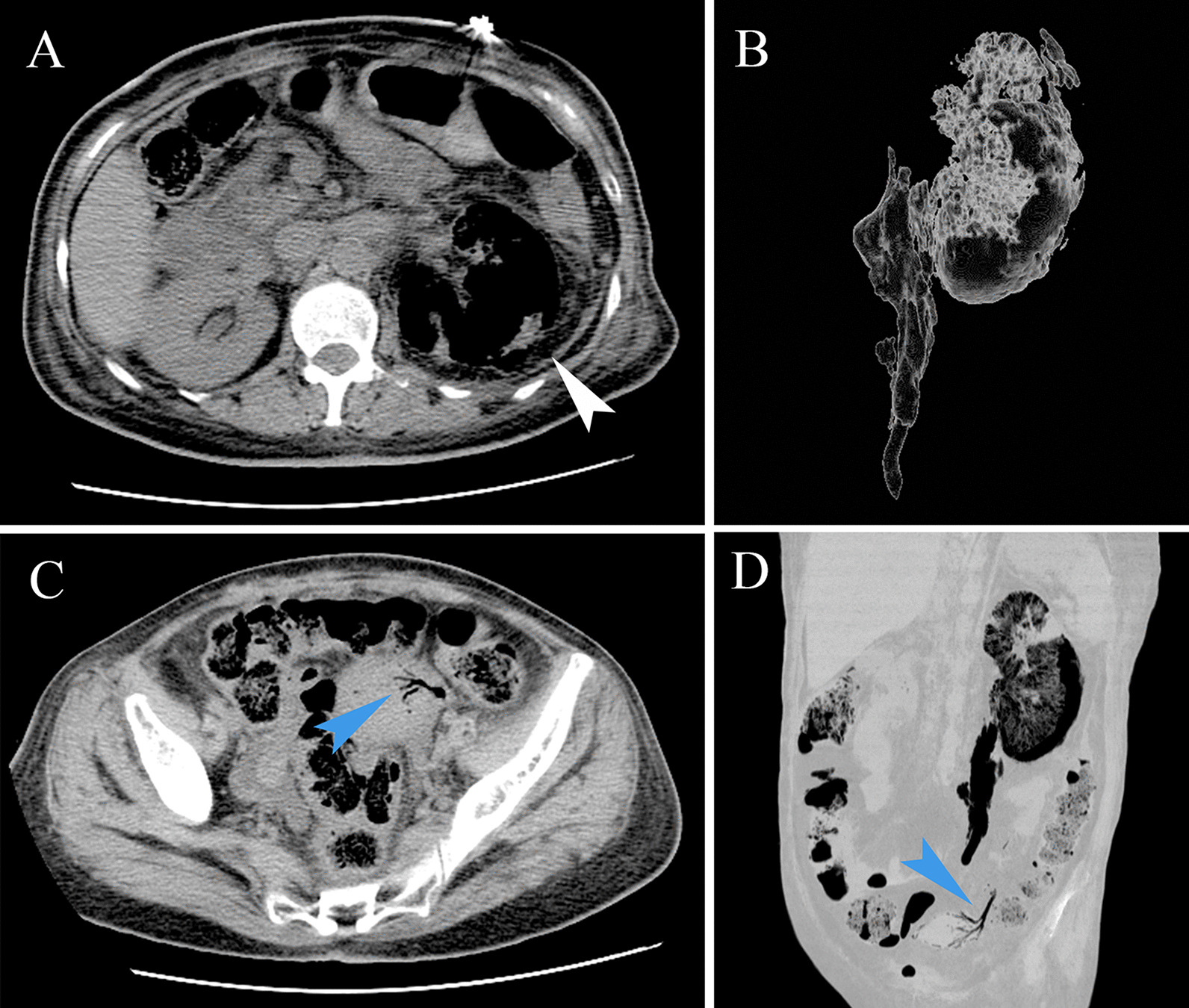
Images in 53-year-old female with emphysematous pyelonephritis (EPN) of the left kidney. **A**, **B** Axial and volume-rendered abdominal CT shows the presence of gas in the parenchyma, para-renal space and left ureteral upper and middle part (white arrow). **C**, **D** Axial and multiplanar reconstruction in minimum-intensity projection CT shows air bubbles in the uterine vein (blue arrow)

An abdominal CT scan showed a diffusely expanded left kidney and ureter with architectural distortion due to linear and columnar streaks of gas in the parenchyma, para-renal space and left ureteral upper and middle part (Fig. 1A, B). There were also air bubbles in the uterine vein which showed “Dead tree branch-like” appearance. (Fig. 1C, D). Based on these findings, the patient was diagnosed with EPN with air in the uterine vein. The blood, pus and urine cultures were positive for extended-spectrum beta-lactamase-producing *E. coli.*

**Fig. 2:**
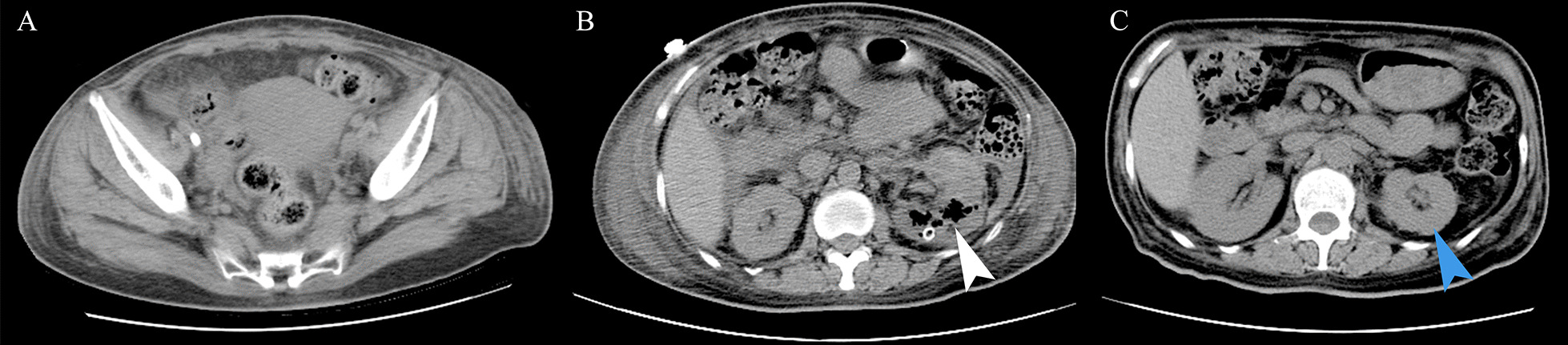
The follow-up computed tomography scan. **A**, **B** Abdominal and pelvic CT taken 3 days after open drainage show reducing of gas the uterine vein and in the left kidney (white arrow). **C** The 3-month follow-up abdominal CT shows complete absence of gas and shrunk-volume in the left kidney (blue arrow)

Percutaneous drainage (PCD) therapy was conducted first to treat the primary lesion and antibiotic treatment was continued. Continuous renal replacement therapy (CRRT) was conducted due to acute renal failure. Due to the poor outcomes of PCD treatment and risk of developing an air embolism, open drainage was performed. Three days after the operation, a repeat CT scan showed reduced gas in the left kidney and uterine vein (Fig. 2A, B). She was closely monitored, and her inflammatory markers and renal function gradually recovered. She was finally discharged 20 days after admission and was instructed to continue her follow-up at the medical office. The abdominal CT, taken 3 months post-discharge, revealed gas disappearance and a shrunk left renal volume (Fig. 2C).

## Discussion and conclusions

EPN is a life-threatening and acute severe necrotizing infection, involving the renal parenchyma, collecting system, and/or perinephric tissue by gas-producing uropathogens. *E. coli* has been isolated from pus or urine cultures in approximately 75% of EPN cases, and other pathogens, such as Klebsiella and Proteus, have also been reported [1]. Its risk factors include DM (up to 95% of patients), female gender, reduced host immunity, chronic urinary tract infections, and genitourinary obstruction [1, 2]. Mortality in EPN has been attributed to patients with disturbed consciousness, thrombocytopenia, and septic complications [1, 3].

Abdominal CT is currently the preferred radiographic modality for diagnosing EPN and staging its severity, which is correlated with its management [4]. EPN is classified into four classes: Class 1 indicates gas confined to the collecting system; Class 2 indicates gas confined to the renal parenchyma without extension to extrarenal space; Class 3A indicates extension of gas or abscess to perinephric space-as in our patient; Class 3B pertains to extension of the gas or abscess to the pararenal space; and Class 4 refers to bilateral EPN or a solitary kidney with EPN [5]. According to this classification, our patient was had Class 3B EPN.

Venous gas is a rare complication of EPN and, to our knowledge, there have been only 15 previously reported cases of EPN with venous gas (Table 1) [6–20]. Of these cases, 13 (87%) of patients were diabetic, and 12 (80%) were female. *E. coli* were the most common bacteria identified (11/15, 73%). The portal vein (7/15, 47%) and the inferior vena cava (5/15, 33%) were the most common blood vessels involved. To our knowledge, there have been no previous reports of EPN with venous gas in the uterine vein. Thrombosis is another rare complication of EPN. According to our knowledge, there have been only three previously reported cases of EPN with venous thrombosis (Table 1) [21–23]. All three cases were female, and unlike venous gas, the renal veins were the most common blood vessels involved by EPN with venous thrombosis.

**Table 1:** Summary of previously published cases of emphysematous pyelonephritis with venous gas/thrombosis

Author (Ref)	Reported Time	Age in years, gender	Diabetesmellitus	Unilateral or Bilateral	Venous Gas/ Thrombosis	Urine culture	Blood culture	Tissue culture	Treatment	Clinical Outcome
^a^Chandra et al. (6)	2020	36, F	Yes	Unilateral	Inferior vena cava	*E. coli*	NA	NA	Antimicrobials + PCD	Improved
^a^Raúl A et al. (7)	2020	56, F	No	Unilateral	Inferior vena cava	Extended-spectrum beta-lactamase-producing *E. coli*	NA	NA	Antimicrobials + nephrectomy	Improved
^a^Tiffany A. et al. (8)	2020	35, F	Yes	Unilateral	Renal vein, inferior vena cava, Pulmonary artery	NA	NA	NA	nephrectomy	Died
^a^Zhong et al. (9)	2019	39, M	Yes	Unilateral	Renal veins	NA	*E. coli*	NA	Antimicrobials + exploratory operation	Improved
^a^Keyvan et al. (10)	2018	64, F	Yes	Unilateral	Vein of the left kidney, pulmonary artery and coronary artery	*Klebsielle pneumoniae*	*Klebsielle pneumoniae*	*Klebsielle pneumoniae*	Antimicrobials + nephrectomy	Improved
^a^Cheng et al. (11)	2015	58, F	Yes	Bilateral	Hepatic portal venous	NA	NA	NA	Antimicrobials	Improved
^a^Mukesh et al. (12)	2014	67, M	NA	Unilateral	Left renal vein	NA	NA	NA	NA	Died
^a^Debraj et al. (13)	2014	65, F	Yes	Unilateral	Hepatic portal venous	NA	NA	*E. coli*	Antimicrobials + nephrectomy	Improved
^a^Jagadish et al. (14)	2011	24, F	Yes	Bilateral	Portal venous system	Imipenem-sensitive *E. coli*	NA	NA	Antimicrobials	Improved
^a^Andrew C et al. (15)	2010	38, F	Yes	Unilateral	Inferior vena cava	Pan-sensitive *E. coli*	Pan-sensitive *E. coli*	NA	Antimicrobials + PCD	Improved
^a^Sung et al. (16)	2010	49, F	Yes	Unilateral	Hepatic portal vein	NA	NA	*E. coli*	Antimicrobials + PCD	Improved
^a^Chang et al. (17)	2009	49, F	Yes	Unilateral	Hepatic portal venous	NA	*E. coli*	*E. coli*	Antimicrobials + PCD	Improved
^a^Mao et al. (18)	2009	56, M	Yes	Unilateral	Hepatic portal venous	NA	*E. coli*	*E. coli*	Antimicrobials + PCD + nephrectomy	Improved
^a^Chen et al. (19)	1994	42, F	Yes	Unilateral	Left renal vein, inferior vena cava and hepatic veins	NA	*E. coli*	*E. coli*	Antimicrobials	Improved
^a^Chang et al. (20)	1992	31, F	Yes	Unilateral	Portal and superior mesenteric venous	NA	NA	*E. coli*	Antimicrobials + PCD	Improved
^b^Jignesh et al. (21)	2021	59, F	Yes	Unilateral	Left renal vein	NA	*Klebsielle pneumoniae*	NA	Antimicrobials + PCD	Improved
^b^Amit Jain et al. (22)	2019	32, F	No	Unilateral	Right renal vein and inferior vena cava	*E. coli*	NA	*E. coli*	Antimicrobials + PCD + nephrectomy	Improved
^b^R.eyaraman et al. (23)	2013	48, F	Yes	Unilateral	Right renal vein and inferior vena cava	*E. coli*	NA	NA	Antimicrobials	Improved

*E. coli* *Escherichia coli*, *F* female, *M* male, *NA* not available, *PCD* percutaneous drainage

^a^Cases of emphysematous pyelonephritis with venous gas

^b^Cases of emphysematous pyelonephritis with venous thrombosis

However, the precise mechanisms of venous air bubble formation in EPN have not been determined. Sebastià et al. [24] proposed three possible mechanisms for the pathogenesis of portomesenteric vein gas associated with infectious abdominal disease: (1) sepsis in the mesentery and portal vein branches; (2) increased intracavitary fermentation of carbohydrates caused by bacteria; and (3) perforation of a mesenteric abscess in the mesenteric lumen, which separates between the peritoneal lobules of the mesenteric membrane to enter the mesenteric vein. It was previously reported that infected bubbles enter the bloodstream and can form seeds in various organs, leading to the spread of infection [12]. In this patient, we speculate that the uterine vein gas was caused by sepsis in the uterine vein, because she had septic shock and a high blood sugar level on admission. The high plasma glucose level serves as a favorable environment for Enterobacteriaceae’s mixed acid fermentation to produce gas, the main components of which are carbon dioxide and hydrogen. The precise mechanisms of venous thrombosis in EPN have also not been determined. Similar to Jignesh et al., we speculate that it was likely provoked by an infectious, and therefore hypercoagulable state [21].

Of 15 patients with venous gas, 5 were treated with nephrectomy, 5 underwent PCD, 3 were treated with antibiotics and general management alone, and 1 patient underwent exploratory operation. Two patients died. One patient, with air in the renal vein and septic emboli in the lungs, died of cardiac arrest within hours of admission. One died because of cardiovascular collapse resulted from septic shock, endogenous air emboli, or a combination of both. Of 3 cases with thrombosis in EPN, one was treated with nephrectomy, one underwent PCD, and one was treated with antibiotics and general management alone. All three patients achieved an improved clinical outcome. However, the incidence of venous gas and thrombosis in patients with EPN may be underestimated, especially in patients with poor prognosis, because venous gas and thrombosis may be overlooked on radiological images of EPN, especially in patients without enhanced abdominal CT.

In Class 3 EPN (based on the Huang and Tseng classification) [5], basic resuscitation and PCD should be performed in conjunction with good glycemic control. Nephrectomy or open drainage should be performed if there is no response to these measures [1]. The clinical presentation in our patient was typical: a diabetic with inadequate glycemic control, vomiting, and disturbance of consciousness. The clinical evolution and CT (Stage 3) led us to perform PCD. The PCD treatment was ineffective, and the EPN was successfully treated by open drainage.

A high degree of clinical suspicion, immediate CT scan, and corresponding medical or surgical treatment, are the basis for successful EPN management. At higher stages (3/4), PCD should be performed as soon as possible, and nephrectomy or open surgery should not be delayed if the PCD is ineffective [3].

In conclusion, EPN requires urgent attention as complications of sepsis can be life-threatening. Venous air bubbles are a rare but potentially fatal complication of EPN, requiring early diagnosis and treatment. Awareness of this disease may lead to early CT scan for diagnosis and staging of disease severity, and may prompt physicians to implement effective antibiotic therapy, PCD or operation to achieve good clinical outcomes.

## Data Availability

The datasets used and analysed during the current study are available from the corresponding author on reasonable request. All authors have read the paper and agree that it can be published.

## Abbreviations

CT: Computed tomography
DM: Diabetes mellitus
*E. coli*: *Escherichia coli*
EPN: Emphysematous pyelonephritis
PCD: Percutaneous drainage
T2DM: Type 2 diabetes mellitus
